# Impact of Personality Trait Interactions on Foraging and Growth in Native and Invasive Turtles

**DOI:** 10.3390/ani14152240

**Published:** 2024-08-01

**Authors:** Lin Gan, Shufang Zhang, Ruyi Zeng, Tianyi Shen, Liu Tian, Hao Yu, Ke Hua, Yue Wang

**Affiliations:** 1 Herpetological Research Center, College of Life Sciences, Nanjing Normal University, Nanjing 210023, China; ganlin@ioz.ac.cn (L.G.);; 2 Center of Reproductive Medicine, Jiaxing Maternity and Child Health Care Hospital, College of Medicine, Jiaxing University, Jiaxing 314000, China; 3 College of Animal Science and Technology, Nanjing Agricultural University, Nanjing 210095, China

**Keywords:** animal personality, invasion dynamics, interspecific competition, foraging strategy, morphology traits, freshwater turtles

## Abstract

**Simple Summary:**

Animal personalities could affect the behavior and physiology of both native and invasive species during biological invasion. However, little is known about the personality interaction effects on foraging behavior and growth between native and invasive species. We used the red-eared slider turtle and Chinese pond turtle as models to investigate how personality interactions effect the foraging behavior and growth of both these turtle species. The results showed that the foraging behavior of *M. reevesii* was mainly affected by the personality of *T. scripta elegans*. However, the foraging behavior of *T. scripta elegans* was effected by both their own personality and the personalities of *M. reevesii.* Additionally, the growth of both *M. reevesii* and *T. scripta elegans* were not effected by the personality combinations. The results revealed the mechanisms of personality interaction effects on the foraging behavior and growth of both native and invasive species during biological invasion. This study provides empirical evidence to help understand the effects of personalities on invasion dynamics.

**Abstract:**

Animal personalities play a crucial role in invasion dynamics. During the invasion process, the behavioral strategies of native species vary among personalities, just as the invasive species exhibit variations in behavior strategies across personalities. However, the impact of personality interactions between native species and invasive species on behavior and growth are rarely illustrated. The red-eared slider turtle (*Trachemys scripta elegans*) is one of the worst invasive species in the world, threatening the ecology and fitness of many freshwater turtles globally. The Chinese pond turtle (*Mauremys reevesii*) is one of the freshwater turtles most threatened by *T. scripta elegans* in China. In this study, we used *T. scripta elegans* and *M. reevesii* to investigate how the personality combinations of native and invasive turtles would impact the foraging strategy and growth of both species during the invasion process. We found that *M. reevesii* exhibited bolder and more exploratory personalities than *T. scripta elegans*. The foraging strategy of *M. reevesii* was mainly affected by the personality of *T. scripta elegans*, while the foraging strategy of *T. scripta elegans* was influenced by both their own personality and personalities of *M. reevesii*. Additionally, we did not find that the personality combination would affect the growth of either *T. scripta elegans* or *M. reevesii*. Differences in foraging strategy may be due to the dominance of invasive species and variations in the superficial exploration and thorough exploitation foraging strategies related to personalities. The lack of difference in growth may be due to the energy allocation trade-offs between personalities or be masked by the slow growth rate of turtles. Overall, our results reveal the mechanisms of personality interaction effects on the short-term foraging strategies of both native and invasive species during the invasion process. They provide empirical evidence to understand the effects of personality on invasion dynamics, which is beneficial for enhancing comprehension understanding of the personality effects on ecological interactions and invasion biology.

## 1. Introduction

The invasion of non-native species is considered a key threat to the integrity of natural habitats and ecosystems worldwide, leading to biodiversity losses [1,2]. Invasive species are known to generate severe economic and environmental harm within native ecosystems [3]. Determining the mechanisms of invasion dynamics and the ecological consequences of non-native species invasion is a crucial question for themes such as invasion ecology, population ecology, and global change ecology [4]. Reptiles, especially freshwater turtles, have become one of the most threatened taxa during biological invasions due to their dependence on freshwater habitats and limited movement abilities [5,6]. Growing evidence shows that invaders are often not a random draw from a population [7,8,9]. Instead, invasive decisions are associated with various ecologically important phenotypic traits, such as personality. For example, bolder and more explorative individuals tend to have a higher rate of success in invading a new habitat [8]. Meanwhile, research has revealed that native species also exhibit individual variations in behavior during biological invasions, which is also related to the various ecologically important phenotypic traits such as personality [10,11].

Animal personality, which refers to an individual’s variety of consistent behavioral differences across time and contexts in a broad range of taxa [12], plays a crucial role in the invasion dynamics of non-native species [13,14,15]. For invasive species, some individuals (e.g., bolder, asocial, more explorative, and active individuals) will colonize empty landscape patches sooner, increasing the probability of an invasive population spreading further, and are more likely to successfully invade new habitats [16,17,18,19]. This variation may be related to their physiological differences, such as body size, energy reserves, and growth rate [20,21]. Moreover, invasive individuals with different personalities respond differently to native species. Some personality types (e.g., bolder, more explorative, and more aggressive) consistently show a greater tendency to compete with native species than others [10,22], which indicates that the reaction of invasive species to native species is also related to personality types. For native species, invasive species have been reported as drivers for behavioral and physiological changes, such as foraging, defending, and growth [23,24,25,26,27,28]. These effects of behavior in native species have been shown to be associated with the personality of invasive species [10,29]. Furthermore, native species with different personalities exhibit differences in behavioral strategies and physiological states in a post-invasion context [10,30]. Even though a growing body evidence has revealed that personality effects the behavior and physiological changes in both native species and invasive species, there is still little known about the impact of personality interaction on behavior and physiological changes between invasive species and native species during a biological invasion. The behavioral and physiological changes when different personalities of invasive species coexist with different personalities of native species remain unclear.

The red-eared slider turtle (*Trachemys scripta elegans*) is native to the southern United States and northern Mexico. It is now becoming invasive in many countries, including China, posing a serious threat to the survival of local native turtle species [31,32]. The *T. scripta elegans* is listed as one of the 100 worst invasive species in the world [33]. The introduction of *T. scripta elegans* would lead to them competing with native freshwater turtle species and lead to the extinction of these native turtles in their native habitats, including species of *Mauremys* [11,32,33]. The native habitat of *T. scripta elegans* is similar to the habitat occupied by the Chinese pond turtle (*Mauremys reevesii*) [34]. Therefore, the *M. reevesii* has become one of the most widely threatened species by *T. scripta elegans* in China. The *M. reevesii* was once the most abundant freshwater turtle in China [35]; however, its wild populations have significantly declined in recent decades and it is currently considered an endangered species [36]. The competition with exotic introduced turtles, mainly the red-eared slider turtle, is one of the major factors responsible for this decline [37,38]. *T. scripta elegans* can outcompete native *M. reevesii* for swimming speed and an advantage in feeding kinematics, such as shorter gape cycle time and neck retraction time [39,40]. These advantages may give the *T. scripta elegans* a competitive edge for food resources, nesting sites, and basking sites [11,32,41]. However, the interaction effects of personality traits in foraging behavior and fitness consequences (e.g., growth) in invasive *T. scripta elegans* and native *M. reevesii* have not been fully studied.

In the current study, we experimentally tested whether there were differences in behavior change and growth of two species when *M. reevesii* with different personalities cohabitated with different personalities of *T. scripta elegans*. Our aim was to investigate the interaction and influence of personality traits between invasive species and native species on behavior and fitness consequences in a post-invasion context. We also aimed to elucidate the relationships among personality, ecological and fitness consequences, and the underlying mechanisms of invasion biology.

## 2. Materials and Methods

### 2.1. Animals

The studied juveniles of *M. reevesii* and *T. scripta elegans* were approximately 14 months old and were purchased from a natural pond-rearing turtle hatchery in Baoying County, Jiangsu Province, China. The turtles were transported to the laboratory and reared in tanks (640 × 430 × 310 mm^3^) filled with tap water (aerated for at least 48 h) with a density of 18 individuals per tank. All turtles were fed a commercial diet (Yahua Ltd., Jining, Shandong, China) once a day at 9:00 a.m. Uneaten food and feces were removed after 1 h, and approximately half of the water was refreshed in the tank each day. All turtles were housed in a room and in a water temperature of 28 ± 1 °C, and they experienced natural photoperiods and an acclimation period of at least three months [42].

### 2.2. Experimental Procedures

After an acclimation period, we used an open-field test and a simulated predator attack test to measure the boldness and exploration personalities of *M. reevesii* (*n* = 49) and *T. scripta elegans* (*n* = 52). To ensure the objectivity of our data, we selected 16 of the relatively most bold exploration turtles and 16 of the relatively most shy avoidance turtles from each species as our focal experimental samples. The number (*n*= 8) of animals in each group was chosen to ensure a statistical significance of the experimental data. Each of the four turtles (randomly selected from the specific personality types) was raised in a tank (520 × 380 × 230 mm^3^) and was randomly marked with 4 colors of non-toxic paint on its shell for individual identification. All turtles lived with their conspecifics and were divided into 4 groups (4 tanks for each group), as follows: (1) the BE-BE group, where two focal turtles with boldness–exploration personalities lived with two other turtles with boldness–exploration personalities; (2) the BE-SA group, where two focal turtles with boldness–exploration personalities lived with two turtles with shy–avoidance personalities; (3) the SA-BE group, where two focal turtles with shy–avoidance personalities lived with two turtles with boldness–exploration personalities; and (4) the SA-SA group, where two focal turtles with shy–avoidance personalities lived with two other turtles with shy–avoidance personalities. After a 4-week acclimatization period [43], we measured the foraging behavior and morphological data of all turtles during the non-invasion stage, where the focal turtles lived with two conspecific turtles. Subsequently, we randomly selected two specific personality *M. reevesii* and two specific personality *T. scripta elegans* reared together in a tank (invasion stage) based on the personality combination groups that developed during the non-invasion stage following a 4-week acclimation period [43]. After this acclimation, we measured their foraging behavior and morphological data again to compare the variations in behavior and growth changes among the species and groups. The foraging behavior was measured in four tanks at a time, and the tanks were selected randomly from each group.

### 2.3. Personality Assay

Both boldness and exploration tests were measured once again two weeks later to determine the consistency and repeatability of boldness–exploration personality traits in turtles.

#### 2.3.1. Boldness Measurement

We following methods described by Kashon and Carlson [44] and Roth et al. [45] to test the turtles’ personalities in terms of boldness. We placed each turtle in the center of a 600 × 600 mm^2^ plastic tank and used a soft rubber rod to gently tap the shell of the turtles to ensure that the head and body retracted into the shell. Researchers then retreated to the test room and monitored the turtles via an overhead camera for 5 min. We recorded the time elapsed from turtles retracting into the shell to (1) head emergence, defined as extending the head from the shell such that the eyes surpassed the anterior margin of the carapace; (2) limbs emergence, defined as extending the limbs from the shell; and (3) movement, when the turtle began walking.

#### 2.3.2. Exploration Measurement

To test exploration personality, we used an open field test to measure turtles’ exploration personality. The open field (600 × 600 × 250 mm^3^) was divided into 4 equal-sized areas. At the beginning of the test, the turtle was placed in the center of the open field and covered by a clear plastic cage for 5 min. Then, we removed the cage and monitored the turtles’ behavior via an overhead camera for 10 min. We used the total number of area crossed and distance moved during the 10 min assay as proxies for exploratory behavior [46,47].

### 2.4. Foraging Behavior

All turtles were given half of their food pellets (approximately 0.5% of body weight) 48 h before the experiment [42] and fasted for 24 h before the experiment. Then, foraging behavior was measured starting at 9:00 a.m. All turtles were given approximately 3% of their body weight in food pellets. We used an overhead camera to monitor the turtles’ behavior for 60 min and then removed any remaining food pellets. The foraging behavior tests were conducted three times during both the non-invasion and invasion stages, with a one-week interval occurring between each test. We recorded the latency, frequency, and duration of foraging during the experiment.

### 2.5. Morphology Traits

Morphological traits included body mass, carapace height, carapace width, and carapace length. Body mass was measured by an electronic balance (Sartorius Model BL 1500, ±0.1 g). Maximum carapace height, carapace straight width, and carapace straight length were measured with a digital caliper (Pro skit, PR China) to the nearest millimeter.

### 2.6. Statistical Analysis

#### 2.6.1. Boldness–Exploration Personality Assessment

We conducted a principal component analysis (PCA) using the “ade4” package in R 4.1.3 to derive composite boldness–exploration scores for each individual [48]. These scores were based on five performance parameters collected during the initial bold and exploration assay [49] and were utilized as individual boldness–exploration personality scores. Subsequently, the second personality assay data were used predict function into the previous PCA model to obtain the second boldness–exploration score. A Markov chain Monte Carlo (MCMC) generalized linear mixed model approach (“MCMCglmm” package in R) was used to assess the repeatability [50]. The repeatability of boldness–exploration personality was evaluated using linear mixed models with the experiment number as a fixed effect, with the turtle’s ID and tank ID acting as random effects. We calculated 95% credible intervals (95% CIs) to assess the repeatability by running 1000 permutations of each test and using the posterior distributions to calculate the repeatability as R = Vind/(Vind + Ve), where Vind = inter-individual variance and Ve = residual variance. The difference in five behavioral parameters between *M. reevesii* and *T. scripta elegans* was analyzed by a one-way ANOVA.

#### 2.6.2. Foraging Behavior

The normality and homogeneity of variance of the foraging behavior data were examined using Shapiro–Wilk and Levene tests, respectively. Due to the non-normal distribution of foraging behavior data, and the presence of zero values, all data were normalized by taking the logarithm base 10 of (x + 1). We conducted a repeated-measures analysis by the GLM procedure to examine the differences in foraging latency, foraging frequency, and foraging duration of *M. reevesii* and *T. scripta elegans* under non-invasion and invasion conditions, followed by Tukey’s post hoc tests. The experimental stage was included as a within-subjects factor. The species of the turtle subjects and the groups were included as two between-subjects factors. Because Mauchly’s test of sphericity for the three behavioral parameters showed *p* < 0.001, the results were reported after a Greenhouse–Geisser correction. Behavioral changes between groups and species were analyzed using a two-way ANOVA followed by Bonferroni correction of post hoc tests. Statistical analyses were performed using the SPSS 20.0 software package.

#### 2.6.3. Morphological Traits

The normality and homogeneity of variance of the morphological traits data were examined using Shapiro–Wilk and Levene tests, respectively. Body mass and carapace height/width ratio were analyzed across experimental stages, species, and groups using a repeated-measures analysis by the GLM procedure followed by Tukey’s post hoc tests. Carapace height, width, and length were analyzed between experimental stages, species, and groups using a repeated-measures analysis by the GLM procedure, with body mass as the covariate, followed by Bonferroni correction post hoc tests. Statistical analyses were performed using the SPSS 20.0 software package.

## 3. Results

### 3.1. Boldness–Exploration Personality

The Principal Component Analysis (PCA) reduced the number of variables to two components with an eigenvalue >1 (Table 1). Together, these components account for 90.11% of the total variance. The PCA results showed that PC1 explained 70.02% of the total variance. It was positively correlated with the exploration test and negatively correlated with the latency measurements of the boldness test. Therefore, we chose to retain the transformed data obtained from PC1 in our study as boldness–exploration scores. The scale of boldness–exploration scores ranged from 3.31 to −3.66. Repeatability analysis revealed that the boldness–exploration personalities of *M. reevesii* and *T. scripta elegans* were highly repeatable. The total repeatability was *R* = 0.567 (95% confidence interval 0.415–0.677), with *R* = 0.540 (95% confidence interval 0.339–0.744) for *M. reevesii* and *R* = 0.610 (95% confidence interval 0.367–0.731) for *T. scripta elegans*.

**Figure 1:**
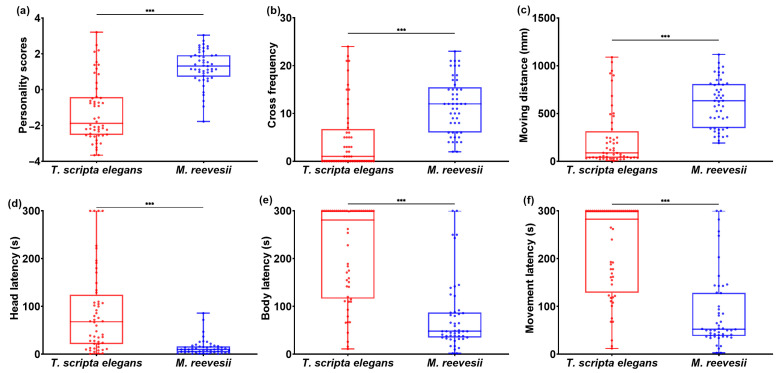
Boldness–exploration personality and behavioral variables difference between *M. reevesii* and *T. scripta elegans*: (**a**) personality scores; (**b**) cross frequency in exploration test; (**c**) moving distance in exploration test; (**d**) head emergency latency in boldness test; (**e**) limbs emergency latency in boldness test; (**f**) movement latency in boldness test. *** *p* < 0.001.

The *M. reevesii* exhibited a significantly higher boldness–exploration score (1.27 ± 0.14) than *T. scripta elegans* (−1.19 ± 0.14) (F_(1, 100)_ = 75.54, *p* < 0.001, as shown in Figure 1a). In terms of behavioral variables, *M. reevesii* exhibited a higher crossing frequency (F_(1, 100)_ = 28.21, *p* < 0.001, Figure 1b) and covered a longer distance while moving (F_(1, 100)_ = 743.14, *p* < 0.001, Figure 1c) than *T. scripta elegans* during exploration tests. *M. reevesii* showed shorter head emergence latency (F_(1, 100)_ = 34.65, *p* < 0.001, Figure 1d), body emergence latency (F_(1, 100)_ = 58.13, *p* < 0.001, Figure 1e), and movement latency (F_(1, 100)_ = 56.62, *p* < 0.001, Figure 1f) than *T. scripta elegans* during boldness tests.

### 3.2. Foraging Behavior

#### 3.2.1. Foraging Latency

**Figure 2:**
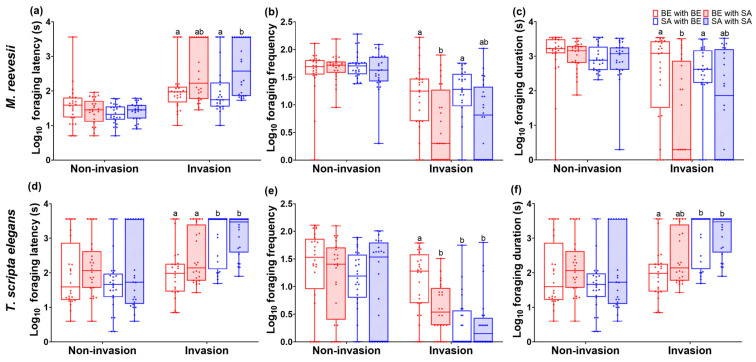
Foraging behavior traits of *M. reevesii* and *T. scripta elegans*: (**a**) log_10_ foraging latency of *M. reevesii*; (**b**) log_10_ foraging frequency of *M. reevesii*; (**c**) log_10_ foraging duration of *M. reevesii*; (**d**) log_10_ foraging latency of *T. scripta elegans*; (**e**) log_10_ foraging frequency of *T. scripta elegans*; (**f**) log_10_ foraging duration of *T. scripta elegans*. Different letters above bars indicate a significant difference in the pairing success rate between combinations (Bonferroni correction of post hoc tests, *p* < 0.05), and the same letter indicates that the difference was not significant at *p* > 0.05).

We found a significant difference in foraging latency between stages (F_(1, 184)_ = 119.49, *p* < 0.001), species (F_(1, 184)_ = 29.64, *p* < 0.001) and groups (F_(1, 184)_ = 5.53, *p* = 0.001). However, there was no interaction effect between species and groups (F_(3, 184)_ = 2.07, *p* = 0.106). The foraging latency between groups of *M. reevesii* showed no significant difference during the non-invasion phase (F_(3, 96)_ = 1.59, *p* = 0.198, Figure 2a). However, there was a significant difference during the invasion phase (F_(3, 96)_ = 4.95, *p* = 0.003, Figure 2a), with the SA-SA group exhibiting a longer foraging latency than the BE-BE and SA-BE groups. The foraging latency between groups of *T. scripta elegans* showed no significant difference during the non-invasion phase (F_(3, 96)_ = 1.15, *p* = 0.333, Figure 2d). However, there was a significant difference during the invasion phase (F_(3, 96)_ = 12.51, *p* < 0.001, Figure 2d), with the SA-BE and SA-SA groups exhibiting longer foraging latency than the BE-BE and BE-SA groups. The foraging latency changes between the non-invasion and invasion phases showed a significant difference for *M. reevesii* and *T. scripta elegans* (F_(1, 64)_ = 4.57, *p* = 0.037). However, there was no significant difference between groups (F_(3, 64)_ = 2.16, *p* = 0.102) and no significant interaction effects between species and groups (F_(3, 64)_ = 0.54, *p* = 0.659).

#### 3.2.2. Foraging Frequency

The foraging frequency showed a significant difference between phase (F_(1, 184)_ = 144.32, *p* < 0.001), species (F_(1, 184)_ = 45.83, *p* < 0.001) and groups (F_(3, 184)_ = 6.86, *p* < 0.001). There was also a significant interaction effect between species and groups (F_(3, 184)_ = 4.98, *p* = 0.002). The foraging frequency between groups of *M. reevesii* showed no significant difference during the non-invasion phase (F_(3, 96)_ = 0.67, *p* = 0.674, Figure 2b). However, there was a significant difference during the invasion phase (F_(3, 96)_ = 4.52, *p* = 0.005, Figure 2b), with the BE-BE and SA-BE groups exhibiting higher foraging frequency than the SA-SA group. The foraging latency between groups of *T. scripta elegans* showed no significant difference during the non-invasion phase (F_(3, 96)_ = 0.75, *p* = 0.524, Figure 2e). However, there was a significant difference during the invasion phase (F_(3, 96)_ = 13.33, *p* < 0.001, Figure 2e), with the BE-BE group exhibiting a higher foraging frequency than the other groups. The foraging frequency changes between the non-invasion and invasion phases were significantly different between *M. reevesii* and *T. scripta elegans* (F_(1, 64)_ = 5.50, *p* = 0.023), but there was no significant difference between groups (F_(3, 64)_ = 1.25, *p* = 0.300) and no significant interaction effects between species and groups (F_(3, 64)_ = 0.50, *p* = 0.684).

#### 3.2.3. Foraging Duration

The foraging duration showed a significant difference between stages (F_(1, 184)_ = 71.04, *p* < 0.001), species (F_(1, 184)_ = 63.53, *p* < 0.001) and groups (F_(3, 184)_ = 7.71, *p* < 0.001). Additionally, there was a significant interaction effect between species and groups (F_(3, 184)_ = 2.82, *p* = 0.040). The foraging duration between groups of *M. reevesii* showed no significant difference during the non-invasion phase (F_(3, 96)_ = 0.31, *p* = 0.815, Figure 2c). However, there was a significant difference during the invasion phase (F_(3, 96)_ = 4.62, *p* = 0.005, Figure 2c), with the BE-BE and SA-BE groups having a longer foraging duration than the BE-SA group. The foraging duration between groups of *T. scripta elegans* showed no significant difference during the non-invasion phase (F_(3, 96)_ = 0.49, *p* = 0.692, Figure 2f). However, there was a significant difference during the invasion phase (F_(3, 96)_ = 9.16, *p* < 0.001, Figure 2f), with the SA-BE and SA-SA groups exhibiting a longer foraging duration than the BE-BE group. The foraging duration changes between the non-invasion and invasion stages were significantly different between *M. reevesii* and *T. scripta elegans* (F_(1, 64)_ = 10.97, *p* = 0.002), but there was no significant difference between groups (F_(3, 64)_ = 2.51, *p* = 0.068) and no significant interaction effects between species and groups (F_(3, 64)_ = 0.20, *p* = 0.099).

### 3.3. Morphology Traits

#### 3.3.1. Body Mass

**Figure 3:**
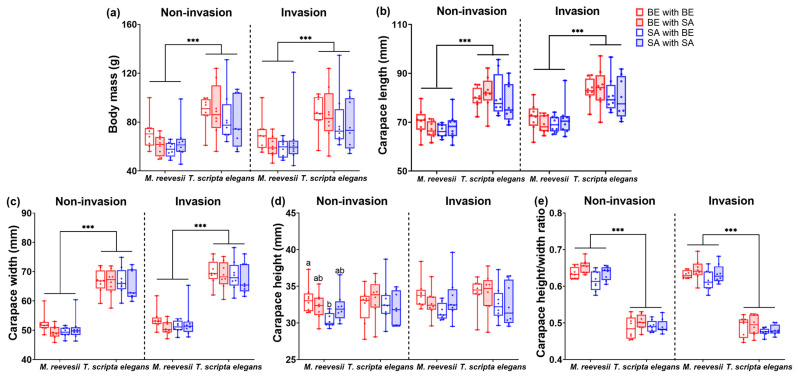
Morphology traits of *M. reevesii* and *T. scripta elegans* between non-invasion and invasion stage: (**a**) body mass; (**b**) carapace length; (**c**) carapace width; (**d**) carapace height; (**e**) carapace height/width ratio. *** *p* < 0.001. Different letters above bars indicate a significant difference in the pairing success rate between combinations (Bonferroni correction of post hoc tests, *p* < 0.05), and the same letter indicates that the difference was not significant at *p* > 0.05).

The body mass showed a significant difference between species (F(_1, 56)_ = 23.89, *p* < 0.001, Figure 3a), with the body mass of *M. reevesii* being lower than that of *T. scripta elegans*. But there was no significant difference between groups (F_(3, 56)_ = 0.87, *p* = 0.464, Figure 3a) and no interaction effect between species and groups (F_(3, 56)_ = 0.47, *p* = 0.706, Figure 3a). Body mass showed a significant difference over time (F_(1, 56)_ = 6.59, *p* = 0.013, Figure 3a), and there was an interaction effect between time and species (F_(1, 56)_ = 7.11, *p* = 0.010). However, there were no interaction effects between time and groups (F_(3, 56)_ = 0.41, *p* = 0.748) or among time, species, and groups (F_(3, 56)_ = 0.76, *p* = 0.524).

#### 3.3.2. Carapace Length

The carapace length showed a significant difference between species (F_(1, 55)_ = 65.79, *p* < 0.001, see Figure 3b), with the carapace length of *M. reevesii* being shorter than that of *T. scripta elegans*. But there was no significant difference between groups (F_(3, 55)_ = 1.03, *p* = 0.388, see Figure 3b) and no interaction effect between species and groups (F_(3, 55)_ = 0.41, *p* = 0.747, see Figure 3b). The carapace length showed a significant difference among time points (F_(1, 55)_ = 6.42, *p* = 0.014, Figure 3b). There was also an interaction effect between time and species (F(_1, 55)_ = 5.42, *p* = 0.024), but no interaction effects were observed between time and groups (F_(3, 55)_ = 0.41, *p* = 0.745) or among time, species, and groups (F_(3, 55)_ = 2.36, *p* = 0.081).

#### 3.3.3. Carapace Width

The carapace width showed a significant difference between species (F_(1, 55)_ = 586.36, *p* < 0.001, Figure 3c), with the carapace length of *M. reevesii* being shorter than that of *T. scripta elegans*. But there was no significant difference between groups (F_(3, 55)_ = 1.11, *p* = 0.354, Figure 3c) and there was no interaction effect between species and groups (F_(3, 55)_ = 0.14, *p* = 0.934, Figure 3c). The carapace width showed a significant difference over time (F_(1, 55)_ = 13.37, *p* = 0.001, see Figure 3c), with no interaction effects being observed between time and species (F_(1, 55)_ = 0.40, *p* = 0.532), time and groups (F_(3, 55)_ = 0.43, *p* = 0.734), or time, species, and groups (F_(3, 55)_ = 2.24, *p* = 0.094).

#### 3.3.4. Carapace Height

The carapace height showed a significant difference between species (F_(1, 56)_ = 34.83, *p* < 0.001, Figure 3d), with the carapace length of *M. reevesii* being shorter than that of *T. scripta elegans*. But there was no significant difference between groups (F_(3, 55)_ = 1.66, *p* = 0.185, Figure 3d) and there was no interaction effect between species and groups (F_(3, 55)_ = 1.27, *p* = 0.292, Figure 3d). The carapace height showed no significant difference over time (F_(1, 55)_ = 2.74, *p* = 0.103, Figure 3d). Additionally, there were no interaction effects observed between time and species (F_(1, 55)_ = 2.25, *p* = 0.139) or between time and groups (F_(3, 55)_ = 1.06, *p* = 0.372). There were interaction effects between time, species, and groups (F_(3, 55)_ = 3.36, *p* = 0.025) The carapace height of the BE-BE group was significantly higher than that of the SA-BE group at the non-invasion stage in *M. reevesii* (*p* = 0.014).

#### 3.3.5. Carapace Height/Width Ratio

The carapace height/width ratio showed a significant difference between species (F_(1, 56)_ = 794.70, *p* < 0.001, Figure 3e), with the carapace length of *M. reevesii* being shorter than that of *T. scripta elegans*, and there was a significant difference between the groups (F_(3, 56)_ = 3.85, *p* = 0.016, Figure 3e), but no interaction effect was observed between species and groups (F_(3, 56)_ = 0.70, *p* = 0.557, Figure 3e). The carapace height/width ratio showed a significant difference over time (F_(1, 56)_ = 6.65, *p* = 0.013, Figure 3e), with no interaction effects between time and species (F_(1, 56)_ = 0.95, *p* = 0.333), time and groups (F_(3, 56)_ = 0.77, *p* = 0.518), or time, species, and groups (F_(3, 56)_ = 1.39, *p* = 0.256).

## 4. Discussion

Animal personalities could affect the behavioral and ecological strategies of both invasive species and native species during the invasion process [13,14,15,51]. Variation in personality affects the strength of selection and the evolution of a species [52,53,54]. Thus, behavioral variations among native species and invasive species may be due to their differences in personalities. The results of the current study show that the native species *M. reevesii* exhibited bolder and more exploratory behavior (1.27 ± 0.14) than the invasive species *T. scripta elegans* (−1.19 ± 0.24), which remained consistent over time. *M. reevesii* moved for longer distances and entered different zones more frequently during the exploration test. Additionally, it exhibited lower hiding behavior in response to modeled predation risk during the boldness test than *T. scripta elegans*. This variation may be due to the potential predator risks in their original habitat. Both *M. reevesii* and *T. scripta elegans* face predation by terrestrial mammals and birds. However, for *T. scripta elegans*, another significant threat comes from aquatic predators, such as caimans and crocodiles [55]. Therefore, it may be safer for *M. reevesii* to emerge from the shell earlier and escape to water because there is no aquatic predation risk in their original habitat [56]. Furthermore, this variation may also impact the difference in body size between the two species. Turtles exhibit a size-dependent hiding response, with larger individuals spending more time concealed inside their shell after an attack before reappearing [57]. This behavior is attributed to the higher costs that small individuals face when hiding, including missed foraging opportunities and increased thermal expenses. Small turtles cannot afford the metabolic expenditure associated with prolonged hiding, as it would detract from foraging time and lead to greater thermal costs due to a faster thermal exchange rate [58,59]. Therefore, the *M. reevesii* exhibited bolder and more exploratory personalities than *T. scripta elegans*. This difference may be an ecological and physiological adaptation consequence and could be crucial for species fitness.

Personality has been well established in regard to having fitness consequences and influencing important life-history decisions, such as foraging strategies [60]. Our results indicate a personality-related variation in foraging behavior between two turtle species. The bolder and more exploratory *M. reevesii* started foraging sooner, with a higher frequency and longer durations, than the shyer and more avoidant *T. scripta elegans*. Earlier and more frequent foraging may help boldness–exploration individuals find more food, but it may also increase the duration of foraging and expose animals to predators [61,62]. The foraging behavior variation between *M. reevesii* and *T. scripta elegans* may result from a trade-off between potential predator risk and physiological requirements. Individuals with poorer body conditions may have higher energy requirements for maintenance, which necessitates putting more effort into foraging and showing a higher motivation for it [21,63,64]. On the other hand, lower foraging effort may indicate a higher foraging efficiency in *T. scripta elegans*. Foraging efficiency is known to be a major determinant of individual fitness. Higher foraging efficiencies mean individuals can gain enough energy in a shorter time, reducing the time exposed to predator risks [65,66]. The larger body size (e.g., gape size) and advantages in feeding kinematics, such as faster movement ability, shorter gape cycle time, and neck retraction time during feeding, would benefit *T. scripta elegans* in completing feeding more quickly than other species [21,39,67]. Therefore, *T. scripta elegans* can forage enough food with a relatively lower foraging effort than other species.

Further, we found that the effects of biological invasions on foraging behavior were more pronounced for *M. reevesii* compared to *T. scripta elegans*. *M. reevesii* exhibited greater changes in their foraging behavior than *T. scripta elegans*, such as longer foraging latency, lower foraging frequency, and shorter foraging duration. This difference may be attributed to the competitive advantages of *T. scripta elegans* [39,67]. Advantages in competitions would presumably be an attempt to increase the profit of dominant species and reduce the overall foraging success of their competitors [68]. Although competition would increase the energy cost for both species, the disadvantaged species experience much lower energetic gains than the advantaged species [68,69]. Consequently, the subordinate *M. reevesii* tend to adopt avoidance strategies that reduce foraging effort in competitive foraging situations to evade the high energetic costs and low gains associated with losing a competition [21,70].

Moreover, we found that the foraging behavior changes were also correlated with their personality types. There were no variations in foraging behavior among groups in both *M. reevesii* and *T. scripta elegans* during the non-invasion stage. During the invasion stage, both *M. reevesii* and *T. scripta elegans* decreased their foraging efforts. For the native species *M. reevesii*, all groups showed increased foraging latency and decreased foraging frequency and duration. Interestingly, we found that the personalities of *T. scripta elegans* had a greater influence on foraging behavior compared to the personalities of *M. reevesii.* The shy–avoidance *T. scripta elegans* significantly reduced the foraging effort of *M. reevesii* compared to their boldness–exploration counterparts. However, the foraging behavior of *T. scripta elegans* was influenced by both their own personality types and *M. reevesii* personality types. Shy–avoidant *T. scripta elegans* exhibited increased foraging latency and longer foraging duration, as well as decreased foraging frequency compared to boldness–exploration individuals. Additionally, coexisting with the shy–avoidance species *M. reevesii* led to longer foraging latency and duration, as well as lower foraging frequency compared to coexisting with boldness–exploration *M. reevesii*. This variation may be related to the competitive advantages and the exploration–exploitation foraging strategies among personalities [21,71,72,73]. Bolder and more exploratory individuals typically adopt an exploration foraging strategy, which involves exploring relatively “superficially” when foraging, while shyer and more avoidant individuals usually opt for an exploitation foraging strategy which involves exploring relatively “thoroughly” when foraging [72,74,75,76]. The exploitation foraging strategy may increase the rate of competition for the same food resources due to the thorough foraging. This exploitation strategy may bring higher competitive pressure than seen with exploration individuals [76]. In the context of interspecific competition, the subordinate species needs to endure larger costs and much lower gains than the dominant species during foraging competition. The food available for the subordinate species is lower when competing with shy–avoidance competitors compared to boldness–exploration competitors [69,72,77,78]. 

From a net energy gain perspective, reducing the investment in foraging effort is a better trade-off for both the boldness–exploration and shy–avoidance subordinate species. The increased competitive pressures from ‘exploitation’ (shy–avoidance) competitors may further decrease the foraging effort of the subordinate species. Of course, dominant species also need to bear the costs of competition, such as a lower net rate of energy gain from foraging, which requires them to prolong the time spent foraging compared to when there are no inter-specific competitors [69,79]. Due to the intense competition and lower net energy gain rate associated with the exploitation strategy, shy–avoidant individuals need to spend much more time foraging compared to bold–exploratory individuals. Additionally, when competing with shy–avoidant individuals, the foraging time is significantly longer than when competing with bold–exploratory individuals. As competition intensifies, the dominant species will spend a significant amount of time expelling the subordinate species from the food resource [69,80,81]. As a result, the shy–avoidance dominant species would reduce the frequency of foraging more than the boldness–exploration dominant species, and they would also reduce the frequency of foraging more when competing with shy–avoidance competitors than with boldness–exploration competitors.

Even though previous studies have noted that biological invasion could reduce the growth rate of native species [26,82], there have also been many studies reporting differences in growth rates between personality types [83,84]. However, we did not find any effects of either the turtles’ own personality or the personality of their competitors on the growth of both *M. reevesii* and *T. scripta elegans*. The energy allocation model could potentially explain this lack of difference, as the turtles may reduce their energy costs in behaviors such as being active or foraging to meet the energy requirements for growth [85]. A recent meta-analysis revealed that the effects of biological invasion on growth were not seen across all taxa but only in certain species [26]. Another recent study provided evidence that there was no significant difference in the growth rates of *M. reevesii* and *T. scripta elegans* [34]. Therefore, the correlation between biological invasion and growth may not be significant in *M. reevesii* and *T. scripta elegans*. Furthermore, given the short duration of our experiment and the turtles’ lower growth rates, the effects of personality and biological invasion may also be masked by the short period of our study. Additional studies are needed to further investigate this issue.

Our results indicate that personality could affect inter-specific competition during invasion dynamics, as personality drives foraging strategies. The personality types of native species and invasive species have an interaction effect on the foraging strategy of both species and may play a crucial role in invasion speeds and dynamics. Although our study was carried out in a relatively short time, the long-term personality effects in the relationship between native species and invasive species, as well as the population dynamics, remain unclear. Further research is needed to provide deeper insights.

## 5. Conclusions

We found that personality can affect the foraging strategies of animals. Additionally, we also found that, during the invasion of non-native species with overlapping ecological niches, foraging strategies are influenced by the personalities of competitors. However, these effects of competitors’ personalities differed between native and non-native species. The foraging strategy of the native *M. reevesii* is mainly influenced by the personality types of the invasive *T. scripta elegans*, while the foraging strategy of *T. scripta elegans* is affected by both the personality types of the *M. reevesii* and *T. scripta elegans* species. These results suggest that the competitive advantages of invasive species and the foraging strategies among different personalities may play a crucial role in the plasticity of foraging strategies for native and invasive species during invasion dynamics. The foraging strategy of disadvantaged native species is primarily influenced by the competitive pressure from the personality-driven foraging strategy of invasive species. Conversely, the foraging strategy of advantaged invasive species is influenced by both the competitive pressure from the personality-driven foraging strategy of native species and their own personality. However, we did not find personality interaction effects on the growth of both native and invasive species. This result may be attributed to the slow growth of freshwater turtles, which short-term experiments could not differentiate. Alternatively, it could be due to the energetic trade-offs of species, where individuals reduce the energy cost of their behavior to allocate more energy towards growth maintenance. Further research is needed to discuss these results. Overall, our results reveal a personality-driven variation in foraging behavior and reveal the mechanisms of personality interactions in foraging strategies between overlapping ecological niches of native and invasive species during non-native species invasions. Our findings provided empirical evidence of the influence of personality traits on inter-specific competition and foraging strategies during invasion dynamics, which is helpful to enhance understanding of the personality effects on ecological interactions and invasion biology.

## Figures and Tables

**Table 1:** Correlation of each behavior observed during the boldness and exploration tests on *M. reevesii* and *T. scripta elegans* with the components of the principal component analysis (PCA).

Behavioral Variables	Component 1 (PC1)	Component 2 (PC 2)
Boldness: head latency	−0.388	−0.470
Boldness: body latency	−0.490	−0.299
Boldness: movement latency	−0.488	−0.292
Exploration: moving distance	0.442	−0.517
Exploration: cross frequency	0.419	−0.580
Eigenvalue	3.50	1.00
Total variance (%)	70.02%	20.09%

## Data Availability

All data included in this study are available upon request from the corresponding author.
